# Smartphone-Based Digital Phenotyping Across Health Conditions: Scoping Review

**DOI:** 10.2196/84146

**Published:** 2026-03-24

**Authors:** Arlen Dumas, Joanne Hokayem, Georgia Goodman, Krishna Venkatasubramanian, Peter Chai

**Affiliations:** 1 Department of Computer Science and Statistics, University of Rhode Island, 9 Greenhouse Road, Kingston, RI, 02881, United States, 1 401-874-4497; 2 The Fenway Institute, Boston, MA, United States; 3 UCLA (University of California, Los Angeles) Health, Los Angeles, CA, United States

**Keywords:** digital phenotyping, smartphones, digital health, scoping review, health behaviors, mobile phone

## Abstract

**Background:**

Smartphone-based digital phenotyping uses built-in sensors and usage patterns to passively capture behavioral and environmental data relevant to health and has been applied extensively in mental health and chronic disease contexts.

**Objective:**

This review synthesizes studies that use smartphone-based digital phenotyping, defined as approaches that rely exclusively on onboard smartphone sensors to characterize specific health conditions. To our knowledge, this work provides the most comprehensive cross-condition synthesis of smartphone-based digital phenotyping to date, spanning mental health, physical health, and substance use disorders (SUDs), and highlighting common practices, gaps, and opportunities for future research.

**Methods:**

We conducted a scoping review of English-language, peer-reviewed papers published between 2012 and 2025 in Google Scholar, IEEE Xplore, ACM Digital Library, and PubMed using terms such as "mobile sensing" and "digital phenotyping." Eligible papers used onboard smartphone sensors to assess health and went beyond self-report. Studies that did not rely on smartphone auxiliary sensing modalities or digital phenotyping were excluded.

**Results:**

We performed a descriptive synthesis of study characteristics, sensors, and health domains. Of 111 papers identified, 65 met inclusion criteria. Most studies were observational and relied on passive sensing. Sample sizes ranged from fewer than 10 to over 18,000 participants, with a median of 52 (IQR=26‐126). Mental health conditions were most frequently examined, including depression (n=16), bipolar disorder (n=11), stress or anxiety (n=10), and schizophrenia (n=8). Less commonly studied conditions included SUDs (n=7), Parkinson disease (n=4), and sleep apnea (n=2). Sensor streams varied widely and included diverse passive smartphone data sources capturing mobility, communication, device usage, environmental context, and user interaction patterns. Ground-truth measurements most commonly relied on validated clinical scales (eg, Patient Health Questionnaire-9, Young Mania Rating Scale [YMRS], and Pittsburgh Sleep Quality Index; n=41), followed by ecological momentary assessments (n=18), clinician-confirmed diagnoses (n=9), and physiological measures such as polysomnography (n=3). Across studies, recurring methodological limitations included incomplete or inconsistent sensor descriptions, limited reporting of data quality (eg, sampling rates and missingness), and heterogeneous validation practices. These issues limit comparability and reproducibility and underscore the need for clearer reporting standards and greater data availability.

**Conclusions:**

This scoping review provides the first comprehensive synthesis of smartphone-only digital phenotyping studies spanning mental health, physical health, and SUDs. Unlike prior reviews, this work maps behavioral associations derived exclusively from smartphone sensors across a broad range of health domains. The primary contribution of this review lies in its consolidation of behavioral associations observed across studies, enabling researchers to correlate new findings to the existing evidence base and identify opportunities for replication, extension, or clinical translation. Collectively, these findings highlight both the promise of smartphone-based digital phenotyping in real-world settings and the need for improved standardization to support translation into clinical and public health applications.

## Introduction

Since their introduction in 1994, smartphones have penetrated almost every aspect of human life worldwide [1]. These devices are ubiquitous in many cultures and have transformed access to the internet and communication with others. Like many other sectors, health care has been directly impacted by the use of smartphones [2,3]. Patients can now conduct virtual visits with their health care providers [4], have access to a wide range of sources of medical information, and can access health records on smartphones [3]. Recent survey data in the United States suggests that 62% of individuals would like to access their medical records through mobile-facing apps, and in 2022, 48% of patients accessed medical records via the internet at least once [5]. Additionally, increased use of wearable and other health-related tracking devices has positioned smartphones as a centralized hub for health data and behavior tracking [6,7]. This data suggests that individuals access and use smartphones to assess and modulate individual health, positioning these devices as important platforms to interact with individuals [6,8].

One potential strategy to leverage smartphones to understand health behavior is through digital phenotyping, a strategy that leverages data from mobile computing devices that most people now have with them (eg, smartphones, smartwatches, rings, and other wearable devices) to capture a person’s behavior, and through them, understand any health conditions present [9]. More specifically, digital phenotyping is the collection and analysis of data generated through human interaction with mobile computing devices. It encompasses (1) passive input collected from sensors on a smartphone, such as continuous GPS monitoring, accelerometry data; and (2) active input, such as ecological momentary assessments (EMAs) completed by the user of the smartphone. By integrating such passive and active inputs, digital phenotyping aims to explore behaviors that correlate with specific health conditions [10].

Smartphone-based digital phenotyping is a form of digital phenotyping in which smartphones are the only devices used for collecting sensor data from individuals. Smartphones offer a unique advantage due to their widespread use, enabling researchers to collect a rich, high-volume dataset that can be used to assess behaviors (also known as phenotypes) in the context of a specific health condition in an unobtrusive way [11]. Data that has been captured via smartphones can be analyzed through a variety of methods to provide insight into a variety of mental and physical health conditions [12]. Compared to traditional approaches of monitoring and diagnosing complex health conditions (eg, schizophrenia and bipolar disorder) that require considerable human interventions, smartphone-based digital phenotyping offers an opportunity to passively track these conditions over time [13]. The other potential advantage of smartphone-based digital phenotyping is the opportunity to democratize diagnoses of various health conditions simply by assessing smartphone use parameters in the context of the user’s known actions [14]. Despite its promise, multiple challenges exist to operationalize smartphone-based digital phenotyping, account for variability in data, and generalize findings across patient populations [9].

This scoping review aims to comprehensively describe the current state of smartphone-based digital phenotyping work. Our scope is more expansive than existing scoping reviews as it covers any and all health conditions that have been explored as part of smartphone-based phenotyping. By exploring the breadth of health conditions studied by smartphone-based digital phenotyping, we can (1) show the versatility of the smartphone-based digital phenotyping to address the needs of a whole variety of health conditions, and (2) obtain a broader understanding of how smartphone-based digital phenotyping can be used across different clinical contexts. Further, in this work, in keeping with the spirit of digital phenotyping, we describe the relationship between smartphone data and behaviors that are exhibited in the presence of specific medical conditions for the works reviewed. We avoid going into algorithmic or machine learning (ML) methods in this scoping review, as that can sometimes promote the discovery without understanding the mindset when looking at prior work. As most of the studies reviewed are observational, the relationships reported between smartphone-derived behaviors and health conditions should be interpreted as associational rather than causal.

This scoping review aims to synthesize the existing literature on smartphone-only digital phenotyping. Specifically, we focused on studies that exclusively use smartphone sensors to characterize health conditions. Our objectives are to consolidate evidence across health domains, describe how behavioral signals are captured and assessed using different smartphone sensor streams, and to identify recurring methodological limitations and gaps in existing literature. By taking a cross-condition perspective, this review clarifies where evidence is concentrated, where it remains sparse, and how future research can better support replication, comparability, and translation into clinical and public health settings.

## Methods

We conducted a scoping review to understand the current state of smartphone-based digital phenotyping. No review protocol was registered for this study. Methods adhered to PRISMA-ScR (Preferred Reporting Items for Systematic Reviews and Meta-Analyses extension for Scoping Reviews) guidelines [15]. The search strategy was reported in accordance with PRISMA-S (Preferred Reporting Items for Systematic Reviews and Meta-Analyses Literature Search Extension) guidelines [16]. We systematically queried Google Scholar, IEEE Xplore, ACM Digital Library, and PubMed for peer-reviewed papers published between 2012 and October 2025. The revised search strategy yielded approximately 3700 records across the 4 databases before screening. Search terms included "mobile sensing," "digital phenotyping," "smartphone sensing," "personal sensing," and "smartphone assessment." Terms were developed through discussion among this study’s team, consisting of experts in digital phenotyping, digital health technologies, behavioral medicine, and computer science. A sample PubMed search string is as follows: ("digital phenotyping" OR "smartphone sensing" OR "mobile sensing") AND (mental OR behavioral OR health OR monitoring). Full search strategies for each database are provided in Multimedia Appendix 1. In addition to database searching, we used backward reference screening of included papers to identify additional studies; we did not search study registries or gray literature. The search strategy followed PRISMA-S guidelines and was informed by prior reviews in digital phenotyping and mobile sensing. We did not use any standardized search filters.

All records retrieved from databases were exported or saved in PDF format from their respective databases and managed manually. Before formal screening, records were manually reviewed to remove duplicate entries and studies that were clearly outside the scope of the review. A substantial proportion of retrieved records were excluded at this stage due to the use of wearable or auxiliary sensors, non–health-related applications, review papers, study protocols, or a primary focus on algorithmic development rather than health characterization. Following this initial filtering step, 111 records remained and proceeded to formal title and abstract screening. This approach is consistent with PRISMA (Preferred Reporting Items for Systematic Reviews and Meta-Analyses) 2020 guidance and is reflected in the PRISMA flow diagram.

Screening followed a 2-stage process consistent with PRISMA-ScR [15]. Two authors (AD and JH) independently screened a total of 111 retrieved PDF records for relevance to smartphone-based sensing and health-related outcomes. A total of 43 studies were identified from PubMed, 21 from IEEE Xplore, 26 from ACM digital library, and 21 from Google Scholar (N=111). After this initial title and abstract review, the same reviewers examined the full text of the remaining studies to confirm that they met the inclusion criteria, which required the use of smartphone-based sensing and a defined health-related outcome. Screening was performed manually, and any disagreements between reviewers were resolved through discussion until consensus was reached. No formal interrater reliability statistic (eg, Cohen k) was calculated, as the screening process was exploratory and descriptive in nature. Reasons for exclusion at each stage, including the use of auxiliary sensors, EMA-only studies, or the absence of health-related outcomes, are summarized in the Results section.

We included only English-language peer-reviewed papers that (1) primarily used smartphone-based sensor data as the input for digital phenotyping studies, (2) described the use of smartphone-based digital phenotyping with relation to a predescribed health condition. We excluded (1) non-English language literature, (2) conference abstracts, white papers, or other policy papers, (3) papers that described digital phenotyping through the use of smartphones as a platform to exclusively perform EMAs or other surveys without using on-board smartphone sensor data.

To find studies for this scoping review, we first determined the eligibility criteria for our studies. For a work to be considered within our scope for this scoping review, it must meet all three of the inclusion criteria listed here: (1) the studies must use digital phenotyping approaches that exclusively use smartphone-based sensor data as input; (2) the studies must not include approaches that exclusively use smartphones as a method for manual data entry, such as EMAs, surveys, etc; and (3) the studies must use digital phenotyping explicitly in the characterization of one or more well-defined health conditions. This includes studies that looked at monitoring the general well-being of an individual.

We did not include studies in this scoping review that solely focused on algorithmic advances or enhanced methods to capture data without analyzing it in the context of specific health conditions.

Using the keywords listed above, we identified 111 studies, of which 46 did not meet the inclusion criteria. This resulted in 65 studies that form the basis of this review. Table 1 shows the frequency of the health conditions represented. The most commonly studied condition was depression (n=18), followed by bipolar disorder (n=10) and stress (n=9). The least represented conditions were substance use disorder (cannabis; n=1) and attention-deficit/hyperactivity disorder (ADHD, n=1).

Before we proceed further, a note about terminology: in the rest of the paper we use the terms study and studies interchangeably. As each of the 65 studies we looked at in this review reported on 1 study, the 2 terms are synonymous in the context of this study.

**Table 1.** This table depicts the counts of health conditions considered by the studies in this scoping review. For studies that cover multiple health conditions, each condition is counted (ie, studies that consider both anxiety and depression are present in the counts for both conditions).

Condition	Count
Depression	18
Bipolar disorder	10
Stress	9
Schizophrenia	8
Anxiety	7
SUD^a^ - alcohol	5
Parkinson disease	4
Sleep apnea	3
SUD - opioids	2
Well-being	2
ADHD^b^	1
SUD - cannabis	1

a SUD: substance use disorder.

b ADHD: attention-deficit/hyperactivity disorder.

## Results

### Overview

**Figure 1.**
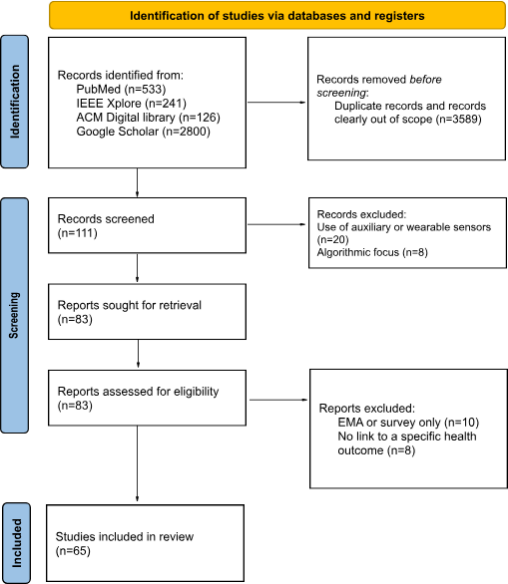
PRISMA flow diagram illustrating the screening and selection process for studies included in this scoping review. The initial search across 4 databases (Google Scholar, IEEE Xplore, ACM Digital Library, and PubMed) identified 111 records published between January 1, 2012, and October 31, 2025. After the removal of nonrelevant records during title and abstract screening, 65 full-text papers met the inclusion criteria for smartphone-based digital phenotyping using on-device sensor data and a defined health-related outcome. Studies were excluded for reasons including use of auxiliary sensors or wearables as primary data sources, EMA-only designs without sensor data, absence of a health-related condition or outcome, or non–peer-reviewed publication type. The final set of 65 studies formed the basis of the analysis presented in this review. EMA: ecological momentary assessment; PRISMA: Preferred Reporting Items for Systematic Reviews and Meta-Analyses.

Before we delve into the details of the findings of our scoping review, we quickly present an overview of prior scoping reviews in the digital phenotyping space and argue why our work is different. We then delve into the findings of our scoping review. Figure 1 depicts a PRISMA flowchart of the review process.

### Prior Scoping Reviews

There are existing reviews that have explored the use of digital phenotyping. Our search yielded 21 prior reviews. We classify these reviews based on the types of sensing technology used: smartphones, smartwatches, and auxiliary sensors. We define auxiliary sensors to be any sensing device outside of smartphones and smartwatches, such as smart textiles or sensors worn on the body. A brief overview of the reviews reported on in this section can be found in Table 2, which we now elaborate on. These prior reviews were not part of the review process but are only presented to show the distinction between the existing reviews and our scoping review.

**Table 2.** Summary of existing digital phenotyping reviews (n=19). This table summarizes prior reviews and reviews examining digital phenotyping across different sensing modalities and health domains. Each citation lists the number of studies covered, primary sensing technologies (eg, smartphones, smartwatches, or auxiliary sensors), publication year, and main health focus.

Citation	Studies (n)	Sensing modalities used	Year	Area of interest
[17]	119	Smartphones	2018	Health and well-being
[18]	18	Smartphones	2019	Bipolar disorder
[19]	224	Smartwatches	2020	Presurgical risk assessment, postsurgical outcome prediction
[20]	62	Smartphones, smartwatches, auxiliary sensors	2021	Bipolar disorder
[21]	92	Smartphones, smartwatches, auxiliary sensors	2021	Depression, bipolar disorder, anxiety, stress
[22]	51	Smartphones, smartwatches	2022	Depression
[23]	46	Smartphones, smartwatches, auxiliary sensors	2022	Machine learning practices
[24]	31	Smartphones, smartwatches	2022	Available datasets and smartphone apps
[25]	81	Smartphones, auxiliary sensors	2023	Parkinson disease, Alzheimer disease
[26]	11	Smartphones, smartwatches, auxiliary sensors	2023	Anxiety
[14]	40	Smartphones	2023	Schizophrenia, depression, general well-being
[27]	29	Smartphones, smartwatches	2023	Mood disorders, schizophrenia-spectrum
[28]	47	Smartphones	2023	Social behavior sensing
[29]	59	Smartphones, smartwatches	2023	Depression, anxiety, stress, schizophrenia, mood, general well-being
[30]	35	Smartphones	2024	Youth depression and anxiety
[31]	24	Smartphones	2024	Machine learning practices
[32]	40	Smartphones	2024	Depression, stress, anxiety
[33]	112	Smartphones	2025	Digital phenotyping pipeline
[34]	16	Smartphones, smartwatches, auxiliary sensors	2025	Stress monitoring
This scoping review	65	Smartphones	2025	Depression, bipolar disorder, schizophrenia, anxiety, stress, general well-being, substance use, sleep apnea, Parkinson disease

Earlier reviews predominantly examined mental health outcomes such as depression, bipolar disorder, and stress, whereas more recent work expanded to neurological, physiological, and multimodal applications. The final row (gray) highlights the present scoping review, which uniquely synthesizes 65 smartphone-only studies spanning 9 health domains. About a third of the reviews (n=7) focus exclusively on using smartphones for digital phenotyping. Three of these reviews focus on describing phenotyping in the context of specific health conditions: depression [31], bipolar disorder [18], and a combination of depression, stress, and anxiety [32]. The remaining reviews include a scoping review of 35 studies on depression and anxiety in youth [30], a review of 47 studies using smartphones for social behavior sensing [28], a large review of 112 studies characterizing digital phenotyping pipelines [33], and a systematic review of 119 studies on smartphone-based health sensing [17]. Additionally, Bufano et al [27] report on 29 clinical studies using smartphones for psychiatric relapse monitoring.

Another third of the reviews (n=6) cover a combination of smartphone and smartwatch-based digital phenotyping. Similar to the previous category, 2 of the studies focus on phenotyping work addressing mental health conditions: depression [22] and depression, anxiety, stress, schizophrenia, mood, and general well-being [29]. One review covers a purely smartwatch-based digital phenotyping to assess presurgical risk and postsurgical care [19]. The last review in this category reports on publicly available applications and datasets for digital phenotyping that use both smartphones and smartwatches [24].

The remaining reviews (n=8) report on digital phenotyping using a combination of smartphones, smartwatches, and auxiliary sensors. Like the prior 2 sections, the reviews in this category focused on mental health conditions: Alzheimer disease [25]; depression, bipolar disorder, anxiety, and stress [21]; bipolar disorder [20]; and anxiety [26]. The studies by Dlima et al [23] and Lee et al [14] focus on the use of ML to interpret phenotyping data, while Bufano et al [27] examine combined sensing modalities in psychiatric relapse studies. Most recently, Kallio et al [34] provide a review of approximately 16 studies focused on continuous stress monitoring in cognitively demanding work environments using smartphones, smartwatches, and ambient sensors. Their review emphasizes real-world deployment, unobtrusiveness, and user acceptance, expanding the digital phenotyping literature into long-term occupational health monitoring.

Most of these reviews (n=15) cover research that uses more sensing modalities than those available on smartphones. Those that do cover purely smartphone-based digital phenotyping (n=7) are scoped in terms of specific mental health conditions, youth populations, or derived behavioral features.

Our review is different from these prior reviews for three reasons: (1) it focuses exclusively on smartphone-based digital phenotyping; (2) its scope is much more expansive, in that it covers any and all health conditions (and not just mental health conditions); and (3) it focuses on describing the relationships between behaviors captured through phenotyping in the context of specific health conditions and not on algorithms used to extract the behaviors.

### Findings of This Scoping Review

Our aim is to understand the current state of smartphone-based digital phenotyping search which led to the identification of 111 records. After title or abstract screening, 65 studies met the inclusion criteria and proceeded to full-text review; all 65 were included in the synthesis. Across the 65 included studies, we conducted a descriptive synthesis to summarize the sample sizes, study durations, and participant populations. The median sample size across all studies was 52 participants (IQR=26‐126), with a median study duration of 8 weeks (IQR 3 wk to 6 mo). Studies commonly used multiple different measurements for ground truth. Clinical rating scales (eg, Patient Health Questionnaire-9, Hamilton Depression Rating Scale, YMRS, Generalized Anxiety Disorder-7, and Positive and Negative Affect Schedule) were used in 63% (41/65) studies, EMAs in 28% (18/65) studies, clinician-confirmed diagnoses in 14% (9/65) studies, and physiological validation measures (eg, polysomnography or gait laboratory testing) in 5% (3/65) studies. Each of these studies was additionally described by the study setting. Controlled laboratory studies are conducted under precise conditions. Naturalistic “daily life” studies capture behavior in real-world environments. Hybrid study designs intentionally combine both settings within the same study [35].

One way of examining smartphone-based digital phenotyping research is by considering the conditions that studies target. These works can be grouped into 6 broad categories: mood disorders, stress-related disorders, schizophrenia, disorders from the use of psychoactive substances, nervous system disorders, and general well-being management.

In all of these efforts, the smartphone sensors were used to collect a variety of contextual information about the individual, such as call or text logs, phone or app usage, location, accelerometer, and microphone data. These were used to extract phenotypes, such as social functioning, physical activity, movement patterns, and sleep patterns, which were associated with the specific health condition being considered. These phenotypes are then analyzed using some form of ML algorithm to answer questions about specific health conditions. To build such models, knowing the ground truth of the health condition of the participants is essential. This ground truth was usually assessed using EMAs or an appropriate scale based on the condition being tracked. In our description below, we do not focus on the intricacies of the ML algorithms used, as they are largely similar across most of the studies. Rather, as much as possible, we focus on the explanations that these models offer about the relationship between sensing, phenotypes, and the health condition being considered.

For each health condition we discuss in this section, we categorize the work done in one or more of the following focus categories: monitoring, diagnosis, and intervention. Monitoring involves collecting and analyzing smartphone-based digital phenotypes to track the health condition being considered. Diagnosis uses smartphone-based digital phenotypes to identify the presence of the health condition or risks thereof. Intervention involves implementing strategies or treatments, often as a response to monitoring a health condition. Not every health condition is worked on in all 3 categories.

### Mood Disorders

Mood is defined as a pervasive and sustained feeling that is experienced internally [36]. It impacts nearly all aspects of a person’s behavior externally. Mood disorders are described by marked disruptions in emotions. For example, experiencing severe lows called depression or highs called hypomania or mania [36]. Though there are a variety of mood disorders, smartphone-based digital phenotyping work in the context of mood disorders has targeted only 2 major conditions in this regard: depression and bipolar disorder, which we now describe.

### Depression

Depression (otherwise known as depressive disorder and major depressive disorder) involves low moods and a loss of pleasure or interest in activities over a prolonged period of time [37]. Over the years, studies have shown that passive sensor data collected from smartphones can be associated with depression [38-40]. Much of the digital phenotype effort to explore and characterize depression has focused on 4 types of phenotyping efforts: tracking social interactions, tracking location diversity, vocal characteristics, and tracking sleep quality. For these approaches, the ground truth was collected via EMA or self-reported using pertinent scales, such as the Personal Health Questionnaire [41], the Center for Epidemiological Studies-Depression Scale [42], the Children’s Depression Rating Scale-Revised [43], and the Clinical Global Impressions-Severity Scale [44]. Phenotyping work done in the depression space was primarily focused on the monitoring of depression, with some work in the diagnosis focus category. Table 3 provides a summary of the studies reviewed in this section.

**Table 3.** Summary of smartphone-based digital phenotyping studies focused on depression (n=16). Studies examined depressive symptoms and mood-related behaviors through passive smartphone sensing. Common phenotypes included sociability, mobility, activity, and sleep patterns. Sensors most frequently used were GPS, accelerometer, screen state, and communication logs, with PHQ-9^a^ as the most common ground-truth measure.

Citation	Condition	Phenotypes considered	Sensors used	Ground truth	Participants (n)	Duration	Focus	Study setting
[38]	Depression	Sociability, movement patterns	App use, gyroscope	PHQ-9	558 adults	10.7 days average	Monitoring	Naturalistic setting (app recruitment)
[39]	Depression	Movement patterns	GPS, app logs, call logs, text logs, IMU^b^	PHQ-9	38 adults	2 years	Monitoring	Naturalistic setting (Spain)
[40]	Depression	Movement	GPS, weather, light	DASS^c^, PANAS-X^d^	31 adults	2 weeks	Monitoring	Naturalistic setting (United States)
[45]	Depression	Sociability	App use, call logs, GPS, battery, screen state	CES-D^e^	120 adults	1 month	Monitoring	Naturalistic setting (China)
[46]	Depression	Activity, sociability	GPS, screen state	PHQ-9	40 adults	2 weeks	Monitoring	Naturalistic setting (United States)
[47]	Depression	Sleep, activity, sociability	Call logs, accelerometer, light, GPS, screen state	PHQ-9	47 adults with diabetes	20 weeks	Monitoring	Naturalistic setting (India)
[48]	Depression	Sociability, activity, movement	Call or text logs, GPS, accelerometer, light, screen state	PHQ-9, HAM- D^f^, HAM-A^g^	13 adolescents	8 weeks	Monitoring	Naturalistic setting (United States)
[49]	Depression	Sociability, mobility	GPS, gyroscope, call or text logs	CDRS-R^h^	24 adolescents	8 weeks	Monitoring	Naturalistic setting (South Korea)
[50]	Depression	Activity, movement	GPS	PHQ-9	48 college students	10 weeks	Monitoring	Naturalistic setting (United States)
[51]	Depression	Movement patterns	GPS	PHQ-8^i^	28 adults	2 months	Monitoring	Naturalistic setting (app store recruitment)
[52]	Depression	Activity, sociability	Accelerometer, GPS, WiFi, call or text logs	PHQ-9	126 adults	9 months	Monitoring	Naturalistic setting (app store recruitment)
[53]	Depression	Movement patterns	Accelerometer, GPS	PHQ-9	33 adults	11 weeks	Monitoring	Naturalistic setting (Bangladesh)
[54]	Depression	Activity, sleep disruption	GPS, accelerometer, logs, screen state	Self-reported mood	100 college students	28 days	Monitoring	Naturalistic setting (United States)
[55]	Depression	Sociability	Microphone, text, GPS	PHQ-9	302 college students	6 months	Monitoring, diagnosis	Hybrid setting (United States)
[56]	Depression	Psychological stress	Microphone	BDI^j^	1814 adults	2 years	Monitoring, diagnosis	Naturalistic setting (Japan)
[57]	Depression	Sociability	Microphone, call or text logs, GPS	PHQ-9, GAD- 7^k^	70 college students	28 weeks	Diagnosis	Hybrid setting (United States)

a PHQ-9: Patient Health Questionnaire-9.

b IMU: Inertial measurement unit.

c DASS: Depression Anxiety Stress Scales.

d PANAS-X: Positive and Negative Affect Schedule—Expanded Version.

e CES-D: Center for Epidemiologic Studies Depression Scale.

f HAM-D: Hamilton Depression Rating Scale.

g HAM-A: Hamilton Anxiety Rating Scale.

h CDRS-R: Children’s Depression Rating Scale-Revised.

i PHQ-8: Patient Health Questionnaire-8.

j BDI: Beck Depression Inventory.

k GAD-7: Generalized Anxiety Disorder-7.

Concerning monitoring, one of the most common ways of characterizing depression and depressive episodes is by looking at changes in social interaction. The social interaction of a person can be approximated by measuring the number of incoming calls a person receives as well as the amount of time people spend on social media apps [45]. It was found that a lower volume of incoming calls and a higher frequency of social media app use were indicative of depression, indicating that individuals who were depressed were not communicating with others and were instead turning to their phones as a potential form of self-soothing [45]. Similar findings on the presence of inverse correlation between number and length of phone calls, text messages, and overall phone use patterns and depression were found in 4 different studies [46-48,55]. However, in 1 study, the authors looked at an adolescent population in South Korea (as opposed to adults in the aforementioned work) and found that those with depression received more calls compared to the control participants [49]. Though the study did find that a person spending more time on smartphones may be associated with depression, as mentioned above.

Phone call and text logs as a proxy for social connection were not the only way to infer and characterize depression. Another common approach was to look at location features (based on the GPS and inertial measurement unit). It has repeatedly been found that movement patterns may be a similarly strong proxy to mental state [50,51]. A case in point is a study that looked at individuals with spinal cord injuries. For such individuals, there was a direct correlation between a decrease in the number of locations visited (presumably due to their condition) and depression [58]. This was found to be true not just for adults but also for younger populations as well [48,59]. A similar pattern was also reported in other studies that showed that the variance, number of location clusters, and location entropy all decreased in relation to increased depression scores [50,51]. When the location data was analyzed by making assumptions about the location context, such as duration of time away from home, the information could be used to provide interventions for individuals with depression [52]. Beyond just diversity of location, in 1 study, the researchers found that physical activity in general was associated with depression, with lower physical activity (in terms of distance variance from any starting point, greater resting time, greater time using the phone in a lying position, fewer steps, less exercise, and greater staying at home) being associated with increased depression [53].

A few studies have also looked at using sleeping patterns to characterize depression. Measuring sleep quality from smartphones, unlike wearable devices (eg, smartwatches), is not straightforward. Consequently, researchers often make broad assumptions about the daily routines of a typical person and check to see if smartphone usage patterns can detect a break in this routine. For instance, in one of these studies, a higher number of instances of “screen on” states recorded from 12 AM to 6 AM was interpreted as indicating a lower sleep quality or disturbed sleep schedule in individuals [47]. This pattern of being up at night was found to be associated with the presence of depression. In a similar study on college students, it was found that higher variance in sleep patterns was associated with depression [54].

Concerning diagnosis, in recent years, an interesting and different approach to detecting the presence of depression in individuals has been explored using vocal characteristics. In 2 different studies, the authors looked at changes in vocalization and tone based on features such as vocal fry, vocal jitter, and monotony measured via unscripted and scripted audio recordings to determine the presence of depression [55,57]. A similar study also analyzed voice features during phone calls to determine the presence of depression [56]. In one of the aforementioned studies, the researchers tried to extend their vocal characteristics-based depression models to the most extreme cases where individuals are experiencing suicidal ideation [57]. They found that such models were not sufficient and required additional context to predict suicidal risk [57].

### Bipolar Disorder

Bipolar disorder is a mental health condition that causes individuals to experience extreme mood swings, typically between emotional highs (known as mania or hypomania) and lows (depression) [60]. There are several types of bipolar and related disorders, not all of which require for both manic and depressive episodes to be present, but all of which require clear changes in mood, energy, and activity levels [60]. Though very similar to the phenotypes used in characterizing depression, the way they are used for characterizing bipolar disorder is somewhat different. As discussed in the prior section, we know that it is possible to identify depression through a variety of methods, but it is important to note that depressive episodes in bipolar disorder may not be as intense or last as long [60]. In addition, mixed states (having depressive symptoms and manic symptoms at the same time) and manic episodes are just as important to recognize in individuals with bipolar disorder, meaning that while some techniques may be useful between both disorders, they are not the same disorder and cannot be treated as such. With respect to smartphone-based digital phenotyping, considerable work has also focused on exploring diseases such as bipolar disorder. These efforts have primarily looked at 4 types of phenotyping efforts: tracking phone usage, tracking location diversity and routine, and tracking sleep quality. Further, the ground truth for bipolar disorder was collected via EMA or self-reported using a combination of scales such as Hamilton Depression Rating Scale [61] and YMRS [62]. Phenotyping work in bipolar disorder was primarily focused on the monitoring category, with some work for both diagnosis and intervention. Table 4 provides a summary of the research reviewed in this section.

**Table 4.** Summary of smartphone-based digital phenotyping studies focused on bipolar disorder (n=11). Studies used passive smartphone sensors to assess behavioral and physiological markers associated with bipolar disorder. Commonly examined features included sociability, activity levels, and sleep-related changes. Ground-truth measures primarily relied on clinical scales such as HAM-D^a^, YMRS^b^, and QIDS-SR^c^.

Citation	Condition	Phenotypes considered	Sensors used	Ground truth	Participants (n)	Duration	Focus	Study setting
[63]	BD^d^	Physical activity, sociability	GPS, cell tower pings, accelerometer, call logs, text logs	Daily self-reported mood, HAM-D, YMRS	13 adults with BD	12 months	Monitoring	Naturalistic setting (Germany)
[64]	BD	Sociability	Accelerometer, GPS, call data	HAM-D, YMRS	10 adults with BD	12 weeks	Monitoring	Naturalistic setting (Austria)
[65]	BD	Physical activity, sociability	Screen state, text logs, call logs, cell tower pings	HAM-D, YMRS	29 adults with BD	12 weeks	Monitoring	Naturalistic setting (Denmark)
[66]	BD	Sociability	Call logs, text logs	Self-reported mood, YMRS, HAM-D	26 adults with BD, 12 controls	28 days	Monitoring	Naturalistic setting (United States)
[67]	BD	Movement, activity, sociability	Accelerometer, GPS, WiFi, Bluetooth, call logs	Mood self-report, HAM-D, MSS^e^, ADS^f^	10 inpatients with BD	8 weeks	Monitoring	Naturalistic setting (Austria)
[68]	BD	Activity, movement patterns	GPS, WiFi, cell tower pings	QIDS-SR	29 adults with BD, 20 controls	3 months	Monitoring	Naturalistic setting (United Kingdom)
[69]	BD	Movement, sleep disruption	GPS	ASRM^g^, DASS-21^h^	159 adults with BD	1 year	Monitoring	Hybrid setting (Taiwan)
[70]	BD	Activity, sociability, movement	Accelerometer, microphone, battery, screen state, logs, calls	SRM-5^i^	7 adults with BD	4 weeks	Monitoring	Naturalistic setting (Germany)
[71]	BD	Sociability	Call or text logs, microphone, screen state, accelerometer, cell tower pings	Mood self- report	28 adults with BD	12 weeks	Monitoring, diagnosis	Naturalistic setting (United States)
[72]	BD	Sleep, activity	Accelerometer, light sensor	PSQI^j^	22 adults with BD, 23 controls	3 weeks	Intervention	Hybrid setting (Austria)
[73]	BD	Sociability, stress, sleep	Accelerometer, WiFi, logs, screen state, Bluetooth, microphone	Mood self-report, HAM-D, YMRS	18 adults with BD	5 months	Monitoring, diagnosis, intervention	Naturalistic setting (app store recruitment)

a HAM-D: Hamilton Depression Rating Scale.

b YMRS: Young Mania Rating Scale.

c QIDS-SR: Quick Inventory of Depressive Symptomatology—self-report.

d BD: bipolar disorder.

e MSS: Mania Self-Rating Scale.

f ADS: Common Depression Scale.

g ASRM: Altman Self-Rating Mania Scale.

h DASS-21: Depression Anxiety Stress Scales-21.

i SRM-5: Social Rhythm Metric Scale-5.

j PSQI: Pittsburgh Sleep Quality Index.

Concerning monitoring, like tracking depression, several efforts in recent years have focused on tracking a bipolar individual’s mood over time. This information can be used to determine if a person is having a depressive or manic episode. In this regard, one of the common phenotypes is smartphone use pattern [64,65,71,73]. In 1 study, the researchers used features of phone calls themselves, such as total length, average call length, number of unique calls, total number of calls, etc, along with sounds emanating during a phone call to determine the current mood or change in moods of bipolar individuals [64]. The study indicated the presence of some kind of association between bipolar states and these phone usage features, but those exact associations were not clear. In 2 other similar studies, it was found that severe depressive symptoms of bipolar disorder were associated with longer “screen on” state per day, more received calls per day, fewer outgoing calls per day, fewer answered incoming calls per day, and less movement [65,66]. On the other hand, manic symptoms showed the exact opposite pattern as described above.

Another approach to characterizing bipolar disorder has been to look at movement or physical activity using smartphone-based GPS data [67-69]. In another study, researchers found that physical activity changes as mood states transition from one to another [67]. When transitioning from a manic state to a baseline normal state, physical activity decreases, but it increases when transitioning from a depressed state to baseline [67]. In a study focused on quantifying depressive states in those with bipolar disorder, the researchers derived a variety of features from GPS data from an individual’s smartphone and were able to use them to predict acute depressive states with high accuracy [68].

Concerning diagnosis, in the context of smartphone-based bipolar disorder diagnosis, researchers looked at vocal patterns along with self-reported surveys on a variety of mood and sleep patterns, along with smartphone usage metadata from individuals with bipolar disorder to detect manic and depressive states [71]. Overall, it was found that while it was possible to classify bipolar states on voice features alone, combining different passively sensed and self-report data with voice features creates a more robust model in terms of detecting subtler manifestations of bipolar states (eg, hypomania as opposed to mania) [71]. Another potential indicator of a bipolar person’s mood is in patterns of app use on the smartphone by a bipolar individual [73]. In particular, the way that bipolar individuals interact with their phones has a strong correlation to what moods they are experiencing at the time. For instance, in 1 study, the researchers found that increased social interaction is associated with a decrease in self-reported stress level, irritability, and mixed mood. This indicated that increased social interaction is a good marker for normal social functioning outside of mood swings. Further, patterns in the average number of apps being used can be used to detect the presence of manic or depressive episodes, with a higher number of apps used being positively associated with self-reported stress, and a decrease in the average number of apps used was suggested to indicate a depressive episode [73].

Concerning intervention applications, finally, sleep disturbances have been shown to be early warning signs of manic or depressive episodes in individuals with bipolar disorder [72]. As stated above, smartphone use (screen on or off, and accelerometer readings) can provide insight into lower sleep quality and satisfaction with sleep, alluding to potential sleep disturbances [73]. In a different study, the researchers showed that a smartphone-based app could be a valid tool for measuring sleep onset and wake-up time, to measure sleep disturbances. This information can then be used for the management of the depressive symptoms of bipolar disorder [72].

### Stress-Related Disorders

Stress is the perception of an individual that their environmental taxes them or exceeds their adaptive capacity [74]. Stress-related disorders are maladaptive responses to short- or long-term exposures to physical or emotional stressors. Smartphone-based phenotyping work for stress-related disorders can be broadly categorized into 2 groups: anxiety disorders and stress quantification. We describe these below. Table 5 provides a summary of the reviewed work in this section.

**Table 5.** Summary of smartphone-based digital phenotyping studies focused on anxiety, stress, and schizophrenia (n=18). Studies span sociability, movement or activity, sleep, and psychological stress phenotypes using signals such as accelerometer, GPS, microphone, app logs, and communication metadata. Ground-truth measures include standardized scales, self-reports, and clinical or laboratory validations.

Citation	Condition	Phenotypes considered	Sensors used	Ground truth	Participants (n)	Duration	Focus	Study setting
[75]	Anxiety	Sociability, movement patterns	Accelerometer, call logs, text logs	SIAS^a^, DASS-21^b^, PANAS^c^	59 college students	2 weeks	Monitoring	Naturalistic setting (United States)
[76]	Anxiety	Sociability, physical activity	App log, power level, screen, GPS, call or text logs	SIAS, UCLA-LS^d^	127 adults	30 days	Monitoring	Naturalistic setting (China)
[77]	Anxiety	Sociability, physical activity	Light, accelerometer, gyroscope, app logs	STAI^e^	20 healthy adults	30 days	Monitoring	Hybrid setting (Japan)
[78]	Stress	Movement, activity, sociability, psychological stress	Accelerometer, GPS, microphone, WiFi, call or text logs, app logs	Mood self-report	30 employees	8 weeks	Monitoring	Naturalistic setting (Italy)
[79]	Stress	Sleep, activity, sociability	Microphone, GPS, WiFi, accelerometer, light sensor	PSS^f^, PHQ-9^g^, UCLA-LS	47 young adults	10 weeks	Monitoring	Naturalistic setting (United States)
[80]	Stress	Physical activity, sociability	Accelerometer	OLBI^h^	30 employees	8 weeks	Monitoring	Naturalistic setting (Italy)
[81]	Stress	Sociability	Call or text logs, Bluetooth	Stress self-report	117 graduate students	7 months	Monitoring	Naturalistic setting (United States)
[82]	Stress	Activity, sociability, sleep, movement	Accelerometer, microphone, light, GPS, Bluetooth	PHQ-9, PSS, UCLA-LS	48 students	10 weeks	Monitoring	Naturalistic setting (United States)
[83]	Stress	Activity, sociability, stress, movement	GPS, WiFi, microphone, accelerometer	GPA^i^, stress or mood self-report	48 students	10 weeks	Monitoring	Naturalistic setting (United States)
[84]	Stress	Psychological stress	Keyboard pressure	Mood self-report, event context	11 adults	1 laboratory session	Diagnosis	Controlled laboratory setting (United States)
[85]	Schizophrenia	Sociability	Call logs, text logs, microphone	BPRS^j^, hospital relapse records	61 adults with schizophrenia	1 year	Monitoring	Naturalistic setting (United States)
[86]	Schizophrenia	Activity, sociability	Call logs, text logs, microphone	Social function diary	5 adults with schizophrenia	5 days	Monitoring	Naturalistic setting (location not stated)
[87]	Schizophrenia	Movement patterns	GPS	Self-reported activity or context	85 adults with schizophrenia, 58 controls	7 days	Monitoring	Naturalistic setting (United States)
[88]	Schizophrenia	Sociability	GPS, accelerometer, screen, call or text logs	PHQ-9, GAD-7^k^, self-reported symptoms	45 adults with schizophrenia, 43 controls	3 months	Diagnosis	Naturalistic setting (United States)
[89]	Schizophrenia	Movement, sociability	Accelerometer, GPS, call or text logs, screen, battery	Symptom report	17 adults with schizophrenia	3 months	Diagnosis	Naturalistic setting (United States)
[90]	Schizophrenia	Movement, activity, sociability, sleep	Accelerometer, GPS, app logs, light, call or text logs	Mood self-report	21 adults with schizophrenia	8 months	Diagnosis	Naturalistic setting (United States)
[91]	Schizophrenia	Activity level	Accelerometer	Self-reported activity	50 adults with schizophrenia, 70 controls	6 days	Diagnosis	Naturalistic setting (United States)
[92]	Schizophrenia	Sleep quality	GPS, screen, call or text logs, accelerometer	PSQI^l^, laboratory sleep assessment	17 schizophrenia	3 months	Diagnosis	Naturalistic setting (United States)

a SIAS: Social Interaction Anxiety Scale.

b DASS-21: Depression Anxiety Stress Scales-21.

c PANAS: Positive and Negative Affect Schedule.

d UCLA-LS: University of California, Los Angeles Loneliness Scale.

e STAI: Spielberger State-Trait Anxiety Inventory.

f PSS: Perceived Stress Scale.

g PHQ-9: Patient Health Questionnaire-9.

h OLBI: Oldenburg Burnout Inventory.

i GPA: Grade Point Average.

j BPRS: Brief Psychiatric Rating Scale.

k GAD-7: Generalized Anxiety Disorder-7.

l PSQI: Pittsburgh Sleep Quality Index.

### Anxiety Disorder

Anxiety disorders usually involve a persistent feeling of anxiety or dread, which can interfere with daily life. People living with such disorders experience frequent anxiety for extended periods of time [93]. For these efforts, the ground truth was collected via EMA or self-reported using pertinent scales, such as the General Anxiety Disorder Scale (Generalized Anxiety Disorder-7) [94]. There were relatively fewer studies in this space, all of which focused on monitoring.

Concerning monitoring, in 1 study, researchers showed that social function derived based on basic statistics collected about phone calls made, received, missed, diversity of callers, etc, can be used to detect the presence of social anxiety disorder in an individual [75]. Other studies found that lack of social connection in terms of lower call use and increased social media application use [76], and use of phones in the dark [77] were associated with higher levels of anxiety disorders. Further, days of the week may also be important in recognizing anxiety, as self-reported anxiety appears to increase toward the end of the weekend and decrease toward the end of the week, possibly aligning with perceived stress or uncertainty about workloads during the week [77]. Overall, the results for characterizing anxiety disorders were very similar to characterizing depression, such that discerning between one and the other may be challenging. For instance, researchers showed a weak and positive relationship between the predicted social anxiety disorder symptom and depression when capturing social functioning of an individual based on phone call usage statistics [75].

### Stress Quantification

Psychological stress is another condition that has been targeted as part of smartphone-based digital phenotyping research. Though experiencing psychological stress is not an uncommon condition for most individuals, prolonged or significant stress has been linked to a variety of physical and mental illnesses [78]. Prolonged stress may create a space for burgeoning mental illnesses such as depression and anxiety to flourish [37,78,79]. Characterizing stress typically relies on the fusion of a variety of behavioral information about an individual, such as their movement patterns, physical activity, social interaction, and sleep patterns, much like works described in the section above. Phenotyping work related to stress was in the monitoring focus category.

Concerning monitoring, in looking at monitoring stress, researchers looked at automatic stress detection in completely unconstrained environments. The study showed that it was possible to extract time and frequency domain features from accelerometer data to characterize routine and associate these features with self-reported stress levels [80]. In a similar study, the researchers explored more complex fusion models where the relationship between self-reported daily stress and sensor-derived activity changes, speech duration, changes in location, and sleep patterns was associated with changes in participants’ stress levels over the course of the study [78]. In a similar work, the authors found that speech duration, movement information (measured using GPS and WiFi), and sleep duration were associated with self-reported daily stress levels, while their activity information (measured using an accelerometer) was associated with changes in their subjective loneliness [79]. Another approach to measuring stress proposed the fusion of information from call and text logs (this included observing broader patterns from calls or text, such as percentage of calls done during the night, percentage of initiated calls during the night, text response rate, text response latency, and the percentage of initiated texts) combined with proximity information (collected from Bluetooth signals from neighboring devices) along with weather information (temperatures, precipitation, humidity, visibility, etc) to accurately predict the presence of stress [81].

All these aforementioned works focused on work-related stress. Another group of individuals whose stress has been studied in some detail is college students. In 1 study, the researchers developed an app called StudentLife for continuously sensing the day-to-day impact of college workload on stress, sleep, activity, mood, sociability, mental well-being, and academic performance among a single class of 48 students across a 10-week term [82]. Analysis of the data collected shows that students at the start of the term had high positive affect and conversation levels, low stress, and healthy sleep and daily activity patterns. However, as the term progressed and the workload increased, stress appreciably increased while positive affect, sleep, conversation, and activity dropped off [82]. A follow-up study looked at a more general approach to stress quantification in college students, using a geo-fencing approach on the GPS data from the smartphones carried by students [83]. The various spaces students frequented were designated as “study spaces” and “party locations”; and in each of these locations, the student’s behavior is recorded based on smartphone activity and audio collected from the phone’s microphone. The results indicated that students who reported decreasing stress levels throughout the term (self-reported) obtained higher GPAs [83].

Finally, researchers used keyboard pressure as a proxy for detecting the presence of stress. Their idea was that the increase in muscle tension in response to an individual experiencing stress would lead to increased keyboard pressure when typing. In this study, the researchers asked individuals to recall a stressful and relaxing event in the recent past and write about it using an iPhone with a modified QWERTY keyboard. They then logged the timing, position, and pressure values associated with each keyboard interaction. Subsequently, the differences in typing pressure across relaxed and stressful expressive writing tasks were associated with differences in self-reported stress [84].

### Delusional Disorders

A delusion is a false belief based on an inaccurate interpretation of an external reality despite evidence to the contrary [95]. In the context of delusional disorders, smartphone-based phenotyping work has exclusively focused on schizophrenia. Schizophrenia is a significant and often life-altering mental disorder characterized by episodes, which include persistent delusions and/or hallucinations, disorganized thinking and behaviors, and instances of agitation or negative symptoms. The term negative symptoms describes a lessening or absence of normal behaviors and functions related to motivation and interest, or verbal or emotional expression, which can manifest as blunted affect, reduction in speech, goal-directed activities, motivation, and experience of pleasure [96]. We summarize our findings in this space below. Table 5 provides a summary of the reviewed research in this section.

### Schizophrenia

Typically, smartphone-based digital phenotyping schizophrenia research has focused on identifying predictors of negative symptoms. This is done by tracking data such as call patterns, location, physical activity, and sleep quality. The ground truth in most of these studies was collected via EMA. Phenotyping work with respect to schizophrenia space was in both monitoring and diagnosis focus categories.

Concerning monitoring, relatively few works have looked into applying phenotyping to people with schizophrenia. In 1 study, the researchers used patterns in incoming and outgoing calls and their duration, incoming and outgoing text messages, and captured the frequency and duration of nearby voices captured through a smartphone microphone to determine exactly how much social activity an individual with schizophrenia was engaging in. Overall, the researchers reported that reductions in the number and duration of outgoing calls, as well as the number of text messages, were associated with relapses [85].

In examining negative symptoms of schizophrenia, researchers explored the feasibility of clustering GPS location as a proxy for social functioning and determining where individuals with schizophrenia are spending their time. Subsequently, they added contextual labels to the location clusters to attain a general understanding of social functioning for a specific individual [86]. In a similar study, researchers looked at GPS-derived indicators of location to determine if they could be used as a proxy for negative symptoms. They found that GPS-derived estimations of average distance traveled over time, distance from home, and the percentage of GPS samples at home were found to be highly associated with negative symptoms. Essentially, they found that less mobility was associated with greater negative symptom severity—possibly due to diminished motivation [87].

Concerning diagnosis, methods outside of GPS-based locations have also been explored to determine the level of social functioning among people with schizophrenia. In this case, routines and disturbances within those routines are seen as indicators of a relapse and may be used in the future as a marker for potential intervention [89]. Researchers have looked at smartphone sensor data (eg, call or text logs, phone screen status, accelerometer, and GPS information) as a proxy for social functioning to determine the relationship between measuring social rhythm (using phenotyping) and schizophrenia. They found that increased stability in social rhythm and routine was associated with improved symptom scores in schizophrenia. This also implies that using phenotyping data related to sociability, mobility, and physical activity, as we have described above, in detecting depression, anxiety, and bipolar disorder can all be associated with identifying negative symptoms of schizophrenia [88].

In another study involving adults with schizophrenia, the researchers looked at mobility before a relapse and found that visual hallucinations were associated with a change in sleeping patterns and the number of voice and nonvoice sounds captured, while auditory hallucinations decreased with new places visited and an increase in call duration [90]. Further, compared to individuals who did not require clinical intervention, there was a significantly higher rate of anomalies in the 2 weeks directly preceding the relapse. In a similar study, the researchers looked at movement information using purely accelerometry data from the smartphone to identify negative symptoms of schizophrenia. The findings of the study indicate that smartphone accelerometers, which are markers of movement and vigor (measured via accelerometry), hold promise in detecting negative symptoms of schizophrenia [91]. In an additional study, the researchers explored the use of accelerometer data to estimate sleep duration and sleep quality in individuals with schizophrenia. The results showed that these attributes were reliable to indicate the presence of sleep disturbances, which can be early warning signs for psychosis and relapse [92].

### Disorders From the Use of Psychoactive Substances

A substance use disorder (SUD) is a mental disorder in which a person experiences an inability to control their use of psychoactive substances such as alcohol, cannabis, opioids, etc [97]. Understanding these SUDs is an emerging aspect of smartphone-based digital phenotyping. We group the effort in this space into 2 broad categories: alcohol use, and cannabis and opioid use. As with the previous efforts at phenotyping, these approaches use sensor data available from smartphones and associate behaviors such as social functioning, movement patterns, and physical activity to psychoactive substance use. Table 6 provides a summary of the research reviewed in this section. Phenotyping work in SUDs was in both monitoring and diagnosis focus categories for both alcohol use and cannabis and opioid use.

**Table 6.** Summary of smartphone-based digital phenotyping studies focused on substance use disorders (n=7). Studies examined behavioral and physiological markers related to alcohol, cannabis, and opioid use. Phenotypes primarily involved movement, sociability, and respiratory features derived from accelerometer, GPS, microphone, and app log data. Ground-truth measures included self-reported use, craving reports, and laboratory validation of BAC^a^.

Citation	Condition	Phenotypes considered	Sensors used	Ground truth	Participants (n)	Duration	Focus	Study setting
[98]	SUD^b^–alcohol use	Movement, sociability	Accelerometer, battery, call or text logs, light, WiFi, Bluetooth, GPS, gyroscope, screen state	Self-reported alcohol use	30 adults	28 days	Monitoring	Naturalistic setting (location unclear)
[99]	SUD–alcohol use	Movement, sociability	Accelerometer, GPS, screen state, app logs, call or text logs, keystroke data	Mood and craving self-report	24 adults	30 days	Monitoring	Naturalistic setting (United States)
[100]	SUD–alcohol use	Physical activity	Accelerometer	Alcohol use self-report	7 adults	2 weeks	Diagnosis	Naturalistic setting (United States)
[101]	SUD–alcohol use	Physical activity	Accelerometer, gyroscope	Urine-based BAC	65 adults	1 in-laboratory session (5 h)	Diagnosis	Naturalistic setting (United States)
[102]	SUD–cannabis use	Activity, movement, sociability	GPS, accelerometer, call logs	Acute cannabis intoxication self-report	57 adults	30 days	Monitoring	Naturalistic setting (United States)
[103]	SUD–opioid use	Respiratory changes	Microphone	Simulated overdose events	209 injection events	3‐5 min postevent	Diagnosis	Hybrid setting (United States)
[104]	SUD–opioid use	Movement patterns	GPS	Drug craving self-report	189 adults with OUD^c^	16 weeks	Diagnosis	Naturalistic setting (United States)

a BAC: blood alcohol concentration.

b SUD: substance use disorder.

c OUD: opioid use disorder.

### Alcohol Use

One of the main areas of interest in smartphone-based phenotyping around the use of psychoactive substances is in studying alcohol use. These efforts take 2 basic forms: determining the level of intoxication and predicting cravings. Phenotyping work related to alcohol use falls under the monitoring and diagnosis categories.

Concerning monitoring, using phenotyping to determine how intoxicated an individual is has been the most common form of research in this space. One notable study in this domain looked at a wide range of mobile phone sensor data (eg, accelerometry, keyboard usage, screen state, call or text logs, battery status, etc) from young adults with a history of hazardous drinking over a period of a month. It found that time of day, day of week, screen duration, and frequency of keyboard presses had significant associations with heavy drinking, indicating behavioral changes during these episodes [98].

In a slightly different study, the researchers looked at alcohol craving in individuals with alcohol-associated liver disease via phone call status, movement patterns, phone screen state, and keystroke information. They found that the level of craving was directly proportional to loneliness, stress, and anxiety as measured primarily through a decrease in the entropy of their movement patterns [99].

Concerning diagnosis, a couple of other efforts looked at blood alcohol levels based on gait information inferred from smartphone sensors [100,101]. The researchers conducted these studies in an uncontrolled environment where it was not clear where the phone was located on the individual’s body. These works used accelerometers and gyroscopes, respectively. Overall, they found some positive correlation between gait measured through smartphone sensors and the level of intoxication of the individual.

### Cannabis and Opioid Use

Not much work has been done when it comes to characterizing cannabis and opioid use using smartphone-based phenotyping. The extant work in this space can be grouped into the same 2 categories as the alcohol case: determining the level of intoxication and predicting cravings. Similar to alcohol use, phenotyping work related to cannabis and opioid use fell under the monitoring and diagnosis categories.

Concerning monitoring, for cannabis, researchers have looked at identifying slowed psychomotor function as a result of consuming cannabis. They measured psychomotor function in an individual based on their movement pattern and physical activity measured using GPS, accelerometer, and call logs. They found a smaller travel boundary and lower activity changes at times when the individual felt “high.” Additionally, stronger body movements indicated by changes in the accelerometer may be indicative of impaired motor control or unsteady movements typically associated with higher levels of intoxication [102].

Concerning diagnosis, in a study related to the context of opioid use, the researchers explored the feasibility of using smartphone data to monitor respiratory changes indicative of an impending opioid overdose. They showed that they could convert a phone microphone into a short-range active sonar using frequency shift to measure chest motion and respiration. This work was done in a controlled operating room environment of the operating room, where overdose conditions were simulated; this method showed promising accuracy in identifying respiratory depression [103].

Finally, the increased interest in using smartphones to aid in the understanding and treatment of SUDs has created an opportunity to assess substance craving as reported in 1 study. By looking at the past 5 hours of solely GPS-based movement data from individuals with opioid-use disorder and correlating it with environmental exposure (measured as a function of the affluence of the locations they visited), the researchers could predict craving behaviors up to 90 minutes in the future for both heroin and cocaine. They were even able to predict stress in the individuals (indicated through EMA responses) using this information [104].

### Sleep Disorders

Smartphone-based digital phenotyping research has also specifically looked at sleep disorders. However, it is somewhat limited. The main focus in phenotyping for sleep disorders has been on sleep apnea. Sleep apnea is a common condition in which a person’s breathing stops and starts again throughout the night [105]. This is a potentially serious disorder that not only impacts sleep quality, but it may also lead to increased cardiovascular risk and lead to premature death if not treated correctly [105]. Smartphone-based approaches primarily monitor breathing and snoring patterns using the smartphone microphone. The ground truth for these studies was established by recruiting individuals with previously diagnosed sleep apnea or by conducting polysomnography as part of the study. Table 7 provides a summary of sleep disorder research reviewed in this section.

**Table 7.** Summary of smartphone-based digital phenotyping studies focused on sleep disorders, neurological disorders, ADHD^a^, and general well-being (n=10). Studies in these domains used smartphone sensors to assess sleep quality, neurological motor symptoms, attention patterns, and general well-being. Common sensor modalities included microphones, accelerometers, and gyroscopes, with validation against polysomnogram, clinical scales, or self-reported measures shown to be strongly associated with polysomnography results for central apnea, hypopnea, and obstructive apnea [103].

Citation	Condition		Phenotypes considered	Sensors used	Ground truth	Participants (n)	Duration	Focus	Study setting
[106]	Sleep apnea		Sleep disruption, sleep patterns	Microphone	Polysomnogram taken before the study	55 samples	One 8-hour sleep period	Diagnosis	Hybrid setting (location not stated)
[107]	Sleep apnea		Sleep disruption, sleep patterns	Microphone	Polysomnogram score	55 sleep samples	One 8-hour sleep period	Diagnosis	Controlled laboratory setting (location not stated)
[108]	Parkinson disease		Tremors, FOG^b^	Accelerometer	TUG^c^ test	20 adults with PD^d^	One in-laboratory assessment	Diagnosis	Controlled laboratory setting (location not stated)
[109]	Parkinson disease		FOG	Accelerometer, gyroscope	Heel-mounted foot switch, pressure sensor mat	12 adults with PD, 12 healthy controls	One in-laboratory assessment	Diagnosis	Controlled laboratory setting (Singapore)
[110]	Parkinson disease		Tremors	Accelerometer, gyroscope	UPDRS^e^	23 adults with PD	One in-laboratory assessment	Diagnosis	Controlled laboratory setting (Greece)
[111]	Parkinson disease		Tremors	Accelerometer	Diagnosis of PD or ET^f^, reevaluation after 1 year	17 adults with PD, 16 adults with ET, 12 healthy controls	One in-laboratory assessment	Diagnosis	Controlled laboratory setting (location not stated)
[112]	ADHD		Loss of concentration	Text logs	Self-reported symptoms	2 adults with ADHD, 2 healthy controls	10 weeks	Monitoring	Naturalistic setting (United States)
[113]	General well-being		Movement patterns, physical activity level, sociability	Microphone, accelerometer, GPS, text logs, call logs	Self-reported mood	18,000 adults	3 years	Monitoring	Naturalistic setting (app store recruitment)
[114]	General well-being		Physical activity level, sleep disruption, sociability	Accelerometer, microphone, screen state, battery level	Self-reported physical and mental well-being	27 adults	19 days	Intervention	Naturalistic setting (app store recruitment)

a ADHD: attention-deficit/hyperactivity disorder.

b FOG: freezing of gait.

c TUG: Timed Up and Go.

d PD: Parkinson disease.

e UPDRS: Unified Parkinson Disease Rating Scale.

f ET: essential tremor.

### Sleep Apnea

Traditionally, measuring the presence of sleep apnea requires an overnight stay at a sleep laboratory in order for a formal diagnosis to take place [115]. This stringent requirement may discourage individuals from actively pursuing treatment if they are unsure of the outcome of a sleep study or may perceive it to be too much of a hassle. As a result, smartphone-based digital phenotyping aims to remain as unobtrusive as possible. Phenotyping work for sleep apnea was primarily in the diagnosis focus category.

Concerning diagnosis, in 1 study, the researchers proposed a method that used accelerometer data and compared movements to a threshold to determine the extent of sleep movement. This was combined with snoring-related sound data from the smartphone microphone to determine the quality of sleep, which was divided into 3 levels of apnea: no apnea, mild apnea, and severe apnea [106]. In a similar study, researchers monitored the breathing patterns of a person using a smartphone app placed at the bedside. Their approach includes collecting information on breathing, the sound of snoring, and breathing pauses to determine the presence of sleep apnea and hypopnea, by parsing out white noise and various sounds that occur during the night [107].

The team that developed the means to determine the feasibility of using a smartphone microphone to monitor respiratory changes indicative of an impending opioid overdose also developed a similar approach that turned the microphone into a short-range active sonar to detect chest and abdomen movements. The changes in chest and abdomen movement detected from the reflected sound waves then allowed for apnea detection, which was then shown to be strongly associated with polysomnography results for central apnea, hypopnea, and obstructive apnea [103].

### Neurological Disorders

A neurological disorder is any disorder of the nervous system. Examples of symptoms include paralysis, muscle weakness, poor coordination, loss of sensation, seizures, confusion, pain, and altered levels of consciousness [116]. From a smartphone-based digital phenotyping standpoint, work on neurological disorders has primarily focused on characterizing Parkinson disease. Table 7 provides a summary of the research reviewed in this section.

### Parkinson disease

Parkinson disease is a movement disorder of the nervous system that progressively worsens over time. As neurons in the brain weaken and die, individuals with the disorder begin to endure tremors, impaired balance, stiffness in the limbs and trunk of the body, and difficulty with movement [117]. Like sleep apnea, phenotyping work for Parkinson disease was also primarily in the diagnosis focus category.

Concerning diagnosis, an aspect of Parkinson disease that has been explored in the smartphone-based phenotyping context is the detection of real-time freezing of gait (stopping suddenly while walking) [108]. In a similar study, 20 individuals with Parkinson disease were asked to perform a video-recorded Timed Up and Go test with and without dual-tasks while carrying a smartphone. The video and accelerometer recordings from the smartphone were synchronized in order to assess the reliability of the freezing of gait detection system in comparison to clinician ratings, with reasonable results [117]. In a similar study, the researchers looked at the accuracy of smartphone-based gait analysis using the built-in triaxial gyroscope and accelerometer and validated it against 2 heel contact-based measurements. These findings highlighted that smartphone-based gait analysis could serve as an alternative to conventional gait analysis methods (eg, foot-switch systems or sensor-embedded walkways), particularly when those methods are inconvenient (eg, cost-prohibitive) [109].

Smartphones have been evaluated as a way to measure hand tremor in Parkinson disease patients [110]. Tremors are a common symptom of Parkinson disease. In this study, accelerometers and gyroscopes were used to detect and measure hand tremors, which were then associated with clinical scores in the hand tremor components of the Unified Parkinson Disease Rating Scale. The results suggest a relatively strong correlation between the patients’ Unified Parkinson Disease Rating Scale hand tremor scores and the quantitative measurement derived from smartphones [110]. In another similar study, the goal was to develop a diagnostic test to differentiate between Parkinson disease and essential tremor based on time-frequency differences using smartphone-based accelerometer data without the use of neuroimaging techniques [111]. In this case, the smartphone was placed over the back of the outstretched hand of a person with Parkinson disease while at rest. The results demonstrated that not only was the phone-based approach effective, but it was also able to diagnose the presence of Parkinson disease and essential tremor in several participants, each of whom had not been officially diagnosed [111].

### Other Efforts

A small amount of work has also been done in characterizing ADHD and assessing the general mental health and well-being of individuals [112-114]. Table 7 provides a summary of the research covered in this section. Phenotyping work for ADHD was primarily focused on monitoring, whereas general well-being had work in both monitoring and diagnosis focus categories.

Concerning monitoring, with respect to ADHD, researchers collected text data and associated it with weekly self-report ADHD symptom scores. The ground truth for the ADHD symptoms was self-reported by the participants. Overall, the results of the study indicated that specific ADHD inattention, sluggish cognitive tempo, and hyperactivity symptoms could be predicted fairly accurately using the smartphone data collected from college students in a pilot study [112].

Further, smartphone-based phenotyping also focused on general well-being. These studies aimed to associate self-reported mental well-being with routine captured through a smartphone. This was a particularly large and longitudinal study. Overall, the study found that positive or negative affect for an individual could be predicted using passive sensing data (such as GPS, phone or call logs, accelerometer, and microphone data). These results suggest that such approaches could reduce the need for self-report [113].

Concerning intervention, in the 1 intervention study, researchers deployed an app called BeWell [114]. BeWell monitored user behavior, such as sleep, physical activity, and social interaction, based on accelerometer, screen on or off, charging, and microphone data to give individuals a well-being score. The well-being scores range between 0 and 100 and are calculated for each of the 3 dimensions (sleep patterns, physical activity, and social interaction). A score of 100 indicates the person is matching or exceeding recommended guidelines. The idea is to use these scores to eventually provide feedback to individuals to maintain their well-being over time [114].

### Sensor Modalities Used Across Different Health Domains

**Figure 2.**
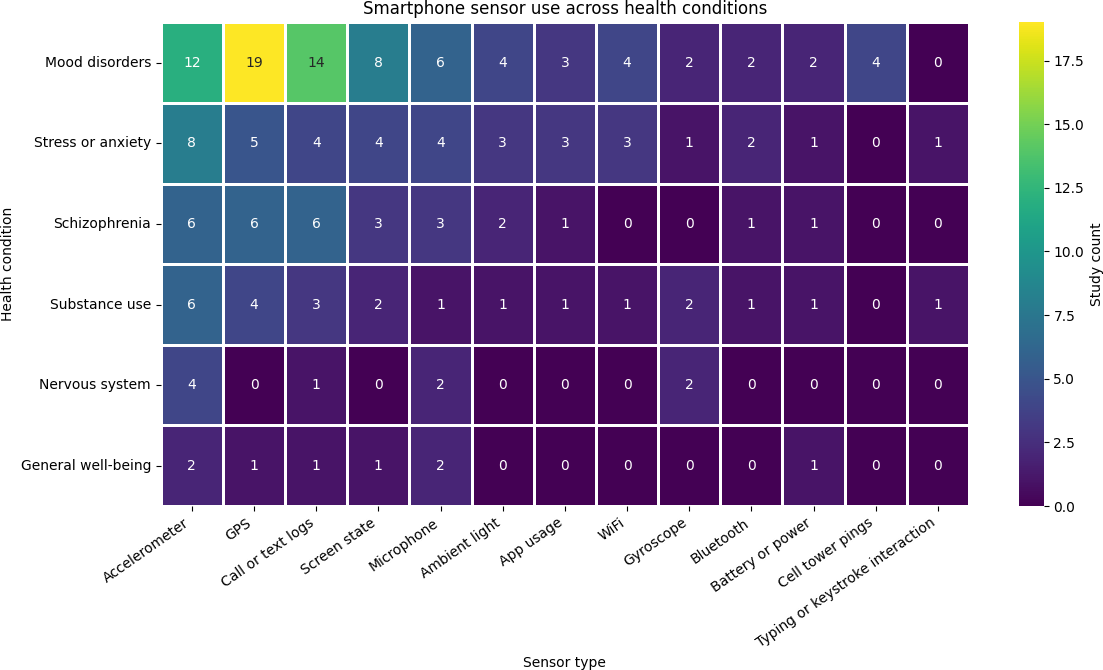
Heatmap illustrating the frequency with which different smartphone sensor modalities were used across 6 major health domains in the reviewed literature. Brighter and warmer-colored cells indicate more frequent usage of a given sensor type within that domain.

Finally, the reviewed studies showed wide variation in sensor use across health domains. While some investigations relied on a single data stream (eg, the work [69] that exclusively used GPS to track bipolar disorder or the research [91] that assessed the activity level of adults with schizophrenia using only the smartphone accelerometer), others combined multiple sources such as GPS, accelerometry, and microphone data to capture a richer behavioral context. As shown in Figure 2, GPS and accelerometer emerged as the 2 most common data streams. Accelerometers appeared as input 58% (38/65) of studies, making them the most frequently used modality overall. GPS followed close behind, appearing in 53% (35/65) studies. Communication logs were also common, with call or text logs appearing in roughly 44% (28/65) of studies. These streams formed the core of most smartphone-based digital phenotyping work due to their ability to intuitively capture mobility, activity, and daily routines. Other smartphone sensing modalities were used far less often. Microphone-based sensing appeared in 27% (18/65) of studies and was fairly evenly spread between health conditions, indicating the potential for widespread use across conditions. Screen state data also appeared in 27% (18/65) of studies, typically as a proxy for phone engagement and sleep-wake cycles. Gyroscope data were used relatively infrequently, appearing in just 10% (7/65) of studies. Environmental and proximity sensing (ambient light, WiFi, cell tower pings, app usage, and Bluetooth) appeared sparingly. At most, these streams appeared in 15% (10/65) of studies in the case of ambient light and just 6% (4/65) of studies in the case of cell tower pings. Battery percentage was used surprisingly infrequently, with only 7% (5/65) of studies using this stream. We speculate this may be due to internal differences in how Android (Google LLC) and iOS (Apple Inc) devices may collect data, but this is not made clear by the authors, who elect to use (or not use) the battery state as a data stream. Keystroke dynamics appeared the least out of all streams being used in just 3% (2/65) of studies. This is likely due to keystroke dynamics being used for specific, tailored tasks as opposed to sensing broad behavioral changes.

## Discussion

### Principal Findings

This scoping review synthesized research that used smartphone-based digital phenotyping to understand health-related conditions. Most studies in our review (n=45) used smartphone-based digital phenotyping to help with mental health conditions. Within the wider health scope, depression and bipolar disorder are of particular interest to researchers due to the ability to effectively track behavioral and mood fluctuations over time through the use of passive smartphone sensors. In contrast, relatively few studies explored other domains such as substance use (n=7), neurological disorders (n=4), or sleep disorders (n=7).

When classified by focus, about 63.5% (41/65) of the studies were conducted with the intent to monitor, which we have defined as tracking symptoms, behaviors, and environmental contexts over time. A smaller subset of these studies (n=15, 23.1%) looked at phenotypes for the purpose of diagnosis, which involves using smartphone-derived features to identify the presence or severity of health conditions. Finally, a few studies totaling 3% (2/65) implemented or evaluated intervention-based approaches in which smartphone-derived behaviors were used to inform treatment strategies or real-time interventions.

Collectively, these findings demonstrate that while smartphone-based phenotyping has some potential across monitoring, diagnostic, and intervention-focused studies for a variety of health conditions, there are several aspects of this research domain that could be improved, which we discuss below.

### Methodological Inconsistencies and Gaps

We found that 15.3% (10/65) of included research studies lacked a clear description of which sensor streams were being used in their phenotyping work and why. This lack of detail about the streams takes several forms: (1) instances where the sensor streams are mentioned as being in scope but are not mentioned in the collection process described [38,39,52,68,112]; (2) instances where a variety of sensor streams are measured from participants but only a subset are used in the phenotyping work [80,92]; and (3) instances where the type of sensor stream collected to capture a phenotype is not even specified [70]. These limitations hinder continued iteration on the described work and suggest the need for guidelines for how to report data surrounding smartphone-based digital phenotyping work to enhance reproducibility, learn from the successes and failures of groups conducting this work, and ultimately advance these tools toward broader impacts. Each of these instances prevents us from fully understanding the scope of the work being done and hinders future research from properly expanding upon the findings of these works. We recommend that, at a minimum, research groups should describe the types of smartphones used in the study, their operating system, the specific sensor streams, and the strategies in which the data was obtained. This methodology could benefit others who seek to leverage smartphone-based digital phenotyping to detect and understand disease trajectories.

These reporting issues extend beyond sensor specifications. Despite all studies using smartphones as the sole sensing device, few provided sufficient technical or procedural detail to allow for replication. Only 9.2% (6/65) of the studies quantitatively reported missingness at the stream level [38,39,52,59,68,92]. Similarly, only 6.1% (4/65) of the studies explicitly reported sampling rates or duty cycles [52,82,83,85], with all other studies leaving the frequency of data capture and transmission unclear. The most common detail reported was phone operating system with 33.8% (22/65) of the studies stating the phone operating system used in data collection, indicating whether the study used Android, iOS, or mixed devices [38,39,48,51-53,59,64,68,70,71,78,80,82,83,85-87,89,90,112,113]. Finally, only 10.7% (7/65) of the studies discussed participant compliance or attrition, offering information about adherence, dropout, or data completeness at the participant level [38,39,52,68,71,82,85].

ML models are commonly used in smartphone-based digital phenotyping work to determine the presence of specific health conditions when monitoring or diagnosing them. Of the 65 studies we assessed as part of this work, about 90.7% (n=59) used ML models, while about 9.3% (n=6) did not [54,72,84,106,109,111]. For our discussion on the use of ML models, we will be noting statistics as being out of 59 so as not to unfairly include studies that do not use ML. About 69.4% (41/59) of the studies that used ML models in their smartphone-based phenotyping work delved deeper into how these models were learning and what derived features may mean in the context of a given health outcome. However, we found that a substantial number, about 30.5% (18/59) of the studies, did not engage with ML models beyond the perfunctory [38,45,53,56,66,69,77,80,81,86,100,101,103,107,108,110,112,118]. These studies reported how accurate these models are with respect to detecting the presence of a health condition in a dataset. While this kind of analysis is important to understanding if models are working, they often eschew any other kind of analysis on why the model is making its decision. We found that these emphases on how accurately an ML model performed did not provide any insight into the validity of the models themselves or an understanding of a person’s behavior being used as phenotypes for characterizing a specific health condition. We believe these points point to a tendency to discover without understanding in a substantial portion of the studies that used ML approaches, which is a cause for numerous errors and bad science [119].

We believe that when ML models are discussed, an effort should be made in exploring the various features used to determine the presence of specific health conditions in addition to reporting on the global accuracy of detection. Reporting on the features allows us, at the very least, to sanity check if the model is making decisions based on known behavioral patterns that individuals with specific health conditions exhibit. However, more importantly, such an analysis has the potential to provide insights into newer ways of identifying these health conditions. This would advance the science of detecting specific health conditions and thus truly leverage the pattern-matching capabilities of ML models.

Finally, we found that despite the amount of work in smartphone-based digital phenotyping, there is a lack of publicly available datasets in this space. The primary issue with not having any data available is that researchers are required to spend a significant amount of time recruiting participants, collecting data, and cleaning data before they perform any kind of analysis. If data were to be made available, innovation and further research would be significantly easier. Only 10.7% (7/65) of studies discuss data availability. Of the studies that provide access to their data, 2 use the Dartmouth StudentLife dataset, which is openly available [82,83]. About 4.6% (3/65) of the studies indicate that the data are available upon request to the authors [49,72,87]. One study indicates that the information is individually identifiable and it must be requested through the institutional ethics board [88]. Only 1.5% (1/65) of the studies provide a working link to the GPS data collected during the study [50]. This suggests that researchers should consider strategies to provide data for other teams to advance smartphone-based digital phenotyping as an innovative strategy to address multiple health conditions. In this process, researchers should consult with their respective institutional ethics boards and ethicists to understand the implications of data sharing, especially for groups whose phenotyping data can be perceived as being more sensitive (eg, specific geolocation and text message data). In some cases, it may be necessary to restrict data access for certain streams of phenotyping data to protect the confidentiality of participants. At the same time, considerations surrounding consent during enrollment for future data sharing to advance the field should be encouraged among research teams. We encourage authors to disclose how to access the data. In the case that data is not readily accessible and must be requested through specific channels, we feel that authors should state this. In case the data is not available under any circumstances, we feel that authors should disclose this as well.

In this scoping review, we provide a comprehensive synthesis of smartphone-based digital phenotyping research across a broader range of health conditions than has been examined in prior reviews. By focusing exclusively on data collected from smartphone sensors, this work differs from existing reviews that either incorporate auxiliary sensing modalities or limit their scope to a small number of predominantly mental health conditions. From a real-world perspective, these results underscore the potential of smartphones as scalable, unobtrusive tools for monitoring, diagnosis, and intervention across mental health, physical health, and SUDs while also highlighting the need for improved reporting practices and validation to support responsible deployment.

### Limitations

This scoping review has a few limitations. First, we excluded non-English and non–peer-reviewed literature, which may have resulted in missing relevant work conducted in other regions or early-stage research not yet published in academic journals. Second, we only searched Google Scholar, IEEE Xplore, ACM Digital Library, and PubMed databases for identifying the studies for this scoping review. Again, searching other databases could have revealed more studies in this space, which we did not include in this review. Third, as a scoping review, we aimed to map available literature to our 3 given focus categories rather than assessing the quality or strength of each study included. As an additional aspect to this point, studies included in this scoping review vary widely in multiple aspects, including the length of the study, the number of participants, and the study setting, which makes direct comparison between studies challenging. Finally, most studies in this review were observational and conducted in relatively small samples, often within specific demographic groups (eg, students). Due to this, the behavioral associations identified should be interpreted as correlational rather than causal, and their applicability to broader or more diverse populations remains an open question.

Despite these limitations, this scoping review provides a comprehensive overview of smartphone-based digital phenotyping across health domains and highlights important gaps that future work must address, particularly around reporting standards, validation practices, and data sharing.

### Conclusion

In this scoping review, we examined the work done in the domain of smartphone-based digital phenotyping in determining the presence of various health conditions. Our scope was much more expansive than prior scoping reviews in this space and includes any and all health conditions that have been explored using smartphone-based phenotyping work. Further, and most importantly, in this work, we focus on describing the relationships between behaviors exhibited in the context of specific health conditions and not on algorithms used to extract these behaviors. Overall, based on a scoping review of 65 studies, we found that smartphone-based digital phenotyping is a powerful and informative tool that can give us insights into a variety of health conditions. Though most of the work has focused on applying smartphone-based digital phenotyping to mental health issues, such as depression, bipolar disorder, and stress, some effort has also gone into phenotyping sleep and neurological conditions.

Smartphone-based digital phenotyping offers substantial promise for understanding health-related behaviors in everyday life. Despite this promise, continued progress depends on addressing key issues surrounding data quality, reproducibility, and reporting standards. Further, as smartphone-based phenotyping scales to larger and more heterogeneous populations, privacy and consent challenges will become increasingly important. Ensuring ethical collection, sharing, and use of sensitive behavioral data will be essential to maintaining participant trust and supporting the responsible growth of this research domain. Smartphones are no longer simple communication tools, but they are emerging as primary instruments for continuous health monitoring.

## Supplementary material

10.2196/84146 Multimedia Appendix 1 Presents the full electronic search strategies used to identify relevant records.

10.2196/84146 Checklist 1 PRISMA-ScR checklist.

## Abbreviations

ADHD: attention-deficit/hyperactivity disorder
EMA: ecological momentary assessment
GAD-7: Generalized Anxiety Disorder-7
ML: machine learning
PRISMA: Preferred Reporting Items for Systematic Reviews and Meta-Analyses
PRISMA-S: Preferred Reporting Items for Systematic Reviews and Meta-Analyses Literature Search Extension
PRISMA-ScR: Preferred Reporting Items for Systematic Reviews and Meta-Analyses extension for Scoping Reviews
SUD: substance use disorder
YMRS: Young Mania Rating Scale

## References

[1] (2021). Mobile technology and home broadband 2021. Pew Research Center.

[2] Ventola CL (2014). Mobile devices and apps for health care professionals: uses and benefits. P T.

[3] Strawley C, Richwine C (2023). ASTP Health IT Data Brief.

[4] Zulman DM, Verghese A (2021). Virtual care, telemedicine visits, and real connection in the era of COVID-19: unforeseen opportunity in the face of adversity. JAMA.

[5] (2019). Trends in individuals’ access, viewing, and use of online medical records and other technology for health needs: 2022. Assistant Secretary for Technology Policy.

[6] Wall C, Hetherington V, Godfrey A (2023). Beyond the clinic: the rise of wearables and smartphones in decentralising healthcare. NPJ Digit Med.

[7] Dorsey ER, Chan YFY, McConnell MV (2017). The use of smartphones for health research. Acad Med.

[8] Ernsting C, Dombrowski SU, Oedekoven M (2017). Using smartphones and health apps to change and manage health behaviors: a population-based survey. J Med Internet Res.

[9] Onnela JP (2021). Opportunities and challenges in the collection and analysis of digital phenotyping data. Neuropsychopharmacology.

[10] Mohr DC, Zhang M, Schueller SM (2017). Personal sensing: understanding mental health using ubiquitous sensors and machine learning. Annu Rev Clin Psychol.

[11] Harari GM, Lane ND, Wang R, Crosier BS, Campbell AT, Gosling SD (2016). Using smartphones to collect behavioral data in psychological science: opportunities, practical considerations, and challenges. Perspect Psychol Sci.

[12] Insel TR (2017). Digital phenotyping: technology for a new science of behavior. JAMA.

[13] Onnela JP, Rauch SL (2016). Harnessing smartphone-based digital phenotyping to enhance behavioral and mental health. Neuropsychopharmacology.

[14] Lee K, Lee TC, Yefimova M (2023). Using digital phenotyping to understand health-related outcomes: a scoping review. Int J Med Inform.

[15] Tricco AC, Lillie E, Zarin W (2018). PRISMA Extension for Scoping Reviews (PRISMA-ScR): checklist and explanation. Ann Intern Med.

[16] Rethlefsen ML, Kirtley S, Waffenschmidt S (2021). PRISMA-S: an extension to the PRISMA Statement for Reporting Literature Searches in Systematic Reviews. Syst Rev.

[17] Cornet VP, Holden RJ (2018). Systematic review of smartphone-based passive sensing for health and wellbeing. J Biomed Inform.

[18] Fraccaro P, Beukenhorst A, Sperrin M (2019). Digital biomarkers from geolocation data in bipolar disorder and schizophrenia: a systematic review. J Am Med Inform Assoc.

[19] Jayakumar P, Lin E, Galea V (2020). Digital phenotyping and patient-generated health data for outcome measurement in surgical care: a scoping review. JPM.

[20] Saccaro LF, Amatori G, Cappelli A, Mazziotti R, Dell’Osso L, Rutigliano G (2021). Portable technologies for digital phenotyping of bipolar disorder: a systematic review. J Affect Disord.

[21] Hilty DM, Armstrong CM, Luxton DD, Gentry MT, Krupinski EA (2021). A scoping review of sensors, wearables, and remote monitoring for behavioral health: uses, outcomes, clinical competencies, and research directions. J Technol Behav Sci.

[22] De Angel V, Lewis S, White K (2022). Digital health tools for the passive monitoring of depression: a systematic review of methods. NPJ Digit Med.

[23] Dlima SD, Shevade S, Menezes SR, Ganju A (2022). Digital phenotyping in health using machine learning approaches: scoping review. JMIR Bioinform Biotech.

[24] Mendes JPM, Moura IR, Van de Ven P (2022). Sensing apps and public data sets for digital phenotyping of mental health: systematic review. J Med Internet Res.

[25] Alfalahi H, Dias SB, Khandoker AH, Chaudhuri KR, Hadjileontiadis LJ (2023). A scoping review of neurodegenerative manifestations in explainable digital phenotyping. NPJ Parkinsons Dis.

[26] Dobson R, Li LL, Garner K, Tane T, McCool J, Whittaker R (2023). The use of sensors to detect anxiety for in-the-moment intervention: scoping review. JMIR Ment Health.

[27] Bufano P, Laurino M, Said S, Tognetti A, Menicucci D (2023). Digital phenotyping for monitoring mental disorders: systematic review. J Med Internet Res.

[28] Zhang H, Ibrahim A, Parsia B, Poliakoff E, Harper S (2023). Passive social sensing with smartphones: a systematic review. Computing.

[29] Moura I, Teles A, Viana D, Marques J, Coutinho L, Silva F (2023). Digital phenotyping of mental health using multimodal sensing of multiple situations of interest: a systematic literature review. J Biomed Inform.

[30] Beames J, Han J, Shvetcov A (2024). Use of smartphone sensor data in detecting and predicting depression and anxiety in young people (12-25 years): a scoping review. SSRN J.

[31] Leaning IE, Ikani N, Savage HS (2024). From smartphone data to clinically relevant predictions: a systematic review of digital phenotyping methods in depression. Neurosci Biobehav Rev.

[32] Choi A, Ooi A, Lottridge D (2024). Digital phenotyping for stress, anxiety, and mild depression: systematic literature review. JMIR mHealth uHealth.

[33] Linardon J, Chen K, Gajjar S (2025). Smartphone digital phenotyping in mental health disorders: a review of raw sensors utilized, machine learning processing pipelines, and derived behavioral features. Psychiatry Res.

[34] Kallio J, Vildjiounaite E, Tervonen J, Bordallo López M (2025). A survey on sensor-based techniques for continuous stress monitoring in knowledge work environments. ACM Trans Comput Healthcare.

[35] Booth BM, Mundnich K, Feng T (2019). Multimodal human and environmental sensing for longitudinal behavioral studies in naturalistic settings: framework for sensor selection, deployment, and management. J Med Internet Res.

[36] Sekhon S, Gupta V (2025). Mood Disorder.

[37] (2025). Depressive disorder (depression). World Health Organization.

[38] Choudhary S, Thomas N, Ellenberger J, Srinivasan G, Cohen R (2022). A machine learning approach for detecting digital behavioral patterns of depression using nonintrusive smartphone data (complementary path to Patient Health Questionnaire-9 assessment): prospective observational study. JMIR Form Res.

[39] Berrouiguet S, Ramírez D, Barrigón ML (2018). Combining continuous smartphone native sensors data capture and unsupervised data mining techniques for behavioral changes detection: a case series of the evidence-based behavior (eB2) study. JMIR mHealth uHealth.

[40] Jacobson NC, Chung YJ (2020). Passive sensing of prediction of moment-to-moment depressed mood among undergraduates with clinical levels of depression sample using smartphones. Sensors (Basel).

[41] Kroenke K, Spitzer RL, Williams JB (2001). The PHQ-9: validity of a brief depression severity measure. J Gen Intern Med.

[42] Radloff LS (1977). The CES-D scale: a self-report depression scale for research in the general population. Appl Psychol Meas.

[43] Poznanski EO, Grossman JA, Buchsbaum Y, Banegas M, Freeman L, Gibbons R (1984). Children’s Depression Rating Scale–Revised. APA PsycTests.

[44] Busner J, Targum SD (2007). The clinical global impressions scale: applying a research tool in clinical practice. Psychiatry (Edgmont).

[45] Wang Y, Ren X, Liu X, Zhu T (2021). Examining the correlation between depression and social behavior on smartphones through usage metadata: empirical study. JMIR mHealth uHealth.

[46] Saeb S, Zhang M, Karr CJ (2015). Mobile phone sensor correlates of depressive symptom severity in daily-life behavior: an exploratory study. J Med Internet Res.

[47] Sarda A, Munuswamy S, Sarda S, Subramanian V (2019). Using passive smartphone sensing for improved risk stratification of patients with depression and diabetes: cross-sectional observational study. JMIR mHealth uHealth.

[48] Cao J, Truong AL, Banu S, Shah AA, Sabharwal A, Moukaddam N (2020). Tracking and predicting depressive symptoms of adolescents using smartphone-based self-reports, parental evaluations, and passive phone sensor data: development and usability study. JMIR Ment Health.

[49] Kim JS, Wang B, Kim M (2023). Prediction of diagnosis and treatment response in adolescents with depression by using a smartphone app and deep learning approaches: usability study. JMIR Form Res.

[50] Saeb S, Lattie EG, Schueller SM, Kording KP, Mohr DC (2016). The relationship between mobile phone location sensor data and depressive symptom severity. PeerJ.

[51] Canzian L, Musolesi M (2015). Proceedings of the 2015 ACM International Joint Conference on Pervasive and Ubiquitous Computing.

[52] Wahle F, Kowatsch T, Fleisch E, Rufer M, Weidt S (2016). Mobile sensing and support for people with depression: a pilot trial in the wild. JMIR mHealth uHealth.

[53] Masud MT, Mamun MA, Thapa K, Lee DH, Griffiths MD, Yang SH (2020). Unobtrusive monitoring of behavior and movement patterns to detect clinical depression severity level via smartphone. J Biomed Inform.

[54] Melcher J, Lavoie J, Hays R (2023). Digital phenotyping of student mental health during COVID-19: an observational study of 100 college students. J Am Coll Health.

[55] Tlachac M, Flores R, Reisch M, Kayastha R, Taurich N, Melican V (2022). StudentSADD: rapid mobile depression and suicidal ideation screening of college students during the coronavirus pandemic. Proc ACM Interact Mob Wearable Ubiquitous Technol.

[56] Higuchi M, Nakamura M, Shinohara S (2020). Effectiveness of a voice-based mental health evaluation system for mobile devices: prospective study. JMIR Form Res.

[57] Tlachac M, Flores R, Toto E, Rundensteiner E (2022). Deep Learning Applications, Volume 4.

[58] Mercier HW, Hamner JW, Torous J, Onnela JP, Taylor JA (2020). Digital phenotyping to quantify psychosocial well-being trajectories after spinal cord injury. Am J Phys Med Rehabil.

[59] MacLeod L, Suruliraj B, Gall D (2021). A mobile sensing app to monitor youth mental health: observational pilot study. JMIR mHealth uHealth.

[60] Bipolar disorder. National Institute of Mental Health.

[61] Hamilton M (1967). Development of a rating scale for primary depressive illness. Br J Soc Clin Psychol.

[62] Young RC, Biggs JT, Ziegler VE, Meyer DA (1978). A rating scale for mania: reliability, validity and sensitivity. Br J Psychiatry.

[63] Beiwinkel T, Kindermann S, Maier A (2016). Using smartphones to monitor bipolar disorder symptoms: a pilot study. JMIR Ment Health.

[64] Grunerbl A, Muaremi A, Osmani V (2014). Smartphone-based recognition of states and state changes in bipolar disorder patients. IEEE J Biomed Health Inform.

[65] Faurholt-Jepsen M, Vinberg M, Frost M (2016). Behavioral activities collected through smartphones and the association with illness activity in bipolar disorder. Int J Methods Psychiatr Res.

[66] Ryan KA, Babu P, Easter R (2020). A smartphone app to monitor mood symptoms in bipolar disorder: development and usability study. JMIR Ment Health.

[67] Grünerbl A, Oleksy P, Bahle G, Haring C, Weppner J, Lukowicz P (2012). Proceedings of the Second ACM Workshop on Mobile Systems, Applications, and Services for HealthCare.

[68] Palmius N, Tsanas A, Saunders KEA (2016). Detecting bipolar depression from geographic location data. IEEE Trans Biomed Eng.

[69] Tseng YC, Lin ECL, Wu CH, Huang HL, Chen PS (2022). Associations among smartphone app-based measurements of mood, sleep and activity in bipolar disorder. Psychiatry Res.

[70] Abdullah S, Matthews M, Frank E, Doherty G, Gay G, Choudhury T (2016). Automatic detection of social rhythms in bipolar disorder. J Am Med Inform Assoc.

[71] Faurholt-Jepsen M, Busk J, Frost M (2016). Voice analysis as an objective state marker in bipolar disorder. Transl Psychiatry.

[72] Fellendorf FT, Hamm C, Dalkner N (2021). Monitoring sleep changes via a smartphone app in bipolar disorder: practical issues and validation of a potential diagnostic tool. Front Psychiatry.

[73] Alvarez-Lozano J, Osmani V, Mayora O, Frost M, Bardram J, Faurholt-Jepsen M (2014). Proceedings of the 7th International Conference on Pervasive Technologies Related to Assistive Environments.

[74] Cohen S, Janicki-Deverts D, Miller GE (2007). Psychological stress and disease. JAMA.

[75] Jacobson NC, Summers B, Wilhelm S (2020). Digital biomarkers of social anxiety severity: digital phenotyping using passive smartphone sensors. J Med Internet Res.

[76] Gao Y, Li A, Zhu T, Liu X, Liu X (2016). How smartphone usage correlates with social anxiety and loneliness. PeerJ.

[77] Fukazawa Y, Ito T, Okimura T, Yamashita Y, Maeda T, Ota J (2019). Predicting anxiety state using smartphone-based passive sensing. J Biomed Inform.

[78] Maxhuni A, Hernandez-Leal P, Morales EF, Sucar LE, Osmani V, Mayora O (2020). Unobtrusive stress assessment using smartphones. IEEE Trans on Mobile Comput.

[79] Ben-Zeev D, Scherer EA, Wang R, Xie H, Campbell AT (2015). Next-generation psychiatric assessment: using smartphone sensors to monitor behavior and mental health. Psychiatr Rehabil J.

[80] Garcia-Ceja E, Osmani V, Mayora O (2016). Automatic stress detection in working environments from smartphones’ accelerometer data: a first step. IEEE J Biomed Health Inform.

[81] Bogomolov A, Lepri B, Ferron M, Pianesi F, Pentland A (2014). MM ’14.

[82] Wang R, Chen F, Chen Z, Li T, Harari G, Tignor S (2014). Proceedings of the 2014 ACM International Joint Conference on Pervasive and Ubiquitous Computing.

[83] Wang R, Harari G, Hao P, Zhou X, Campbell AT (2015). Proceedings of the 2015 ACM International Joint Conference on Pervasive and Ubiquitous Computing.

[84] Exposito M, Hernandez J, Picard RW (2018). Proceedings of the 20th International Conference on Human-Computer Interaction with Mobile Devices and Services Adjunct.

[85] Buck B, Scherer E, Brian R (2019). Relationships between smartphone social behavior and relapse in schizophrenia: a preliminary report. Schizophr Res.

[86] Difrancesco S, Fraccaro P, van der Veer SN (2016). 2016 IEEE 29th International Symposium on Computer-Based Medical Systems (CBMS).

[87] Depp CA, Bashem J, Moore RC (2019). GPS mobility as a digital biomarker of negative symptoms in schizophrenia: a case control study. NPJ Digit Med.

[88] Henson P, Barnett I, Keshavan M, Torous J (2020). Towards clinically actionable digital phenotyping targets in schizophrenia. NPJ Schizophr.

[89] Barnett I, Torous J, Staples P, Sandoval L, Keshavan M, Onnela JP (2018). Relapse prediction in schizophrenia through digital phenotyping: a pilot study. Neuropsychopharmacology.

[90] Wang R, Aung MS, Abdullah S, Brian R, Campbell AT, Choudhury T (2016). Proceedings of the 2016 ACM International Joint Conference on Pervasive and Ubiquitous Computing.

[91] Strauss GP, Raugh IM, Zhang L (2022). Validation of accelerometry as a digital phenotyping measure of negative symptoms in schizophrenia. Schizophrenia.

[92] Staples P, Torous J, Barnett I (2017). A comparison of passive and active estimates of sleep in a cohort with schizophrenia. NPJ Schizophr.

[93] Anxiety disorder. National Institute of Mental Health.

[94] Spitzer RL, Kroenke K, Williams JBW, Löwe B (2006). A brief measure for assessing generalized anxiety disorder: the GAD-7. Arch Intern Med.

[95] Joseph SM (2025). Delusional Disorder.

[96] Correll CU, Schooler NR (2020). Negative symptoms in schizophrenia: a review and clinical guide for recognition, assessment, and treatment. Neuropsychiatr Dis Treat.

[97] Drug addiction (substance use disorder). Mayo Clinic.

[98] Bae S, Ferreira D, Suffoletto B (2017). Detecting drinking episodes in young adults using smartphone-based sensors. Proc ACM Interact Mob Wearable Ubiquitous Technol.

[99] Wu T, Sherman G, Giorgi S (2023). Smartphone sensor data estimate alcohol craving in a cohort of patients with alcohol-associated liver disease and alcohol use disorder. Hepatol Commun.

[100] Arnold Z, Larose D, Agu E (2015). 2015 International Conference on Healthcare Informatics (ICHI).

[101] Li R, Balakrishnan GP, Nie J (2021). Estimation of blood alcohol concentration from smartphone gait data using neural networks. IEEE Access.

[102] Bae SW, Chung T, Islam R (2021). Mobile phone sensor-based detection of subjective cannabis intoxication in young adults: a feasibility study in real-world settings. Drug Alcohol Depend.

[103] Nandakumar R, Gollakota S, Sunshine JE (2019). Opioid overdose detection using smartphones. Sci Transl Med.

[104] Epstein DH, Tyburski M, Kowalczyk WJ (2020). Prediction of stress and drug craving ninety minutes in the future with passively collected GPS data. NPJ Digit Med.

[105] Sleep apnea. Mayo Clinic.

[106] Alqassim S, Ganesh M, Khoja S, Zaidi M, Aloul F, Sagahyroon A (2012). 2012 IEEE 14th International Conference on E-Health Networking, Applications and Services (Healthcom).

[107] Tiron R, Lyon G, Kilroy H (2020). Screening for obstructive sleep apnea with novel hybrid acoustic smartphone app technology. J Thorac Dis.

[108] Capecci M, Pepa L, Verdini F, Ceravolo MG (2016). A smartphone-based architecture to detect and quantify freezing of gait in Parkinson’s disease. Gait Posture.

[109] Ellis RJ, Ng YS, Zhu S (2015). A validated smartphone-based assessment of gait and gait variability in Parkinson’s disease. PLoS ONE.

[110] Kostikis N, Hristu-Varsakelis D, Arnaoutoglou M, Kotsavasiloglou C (2014). Smartphone-based evaluation of parkinsonian hand tremor: quantitative measurements vs clinical assessment scores. Annu Int Conf IEEE Eng Med Biol Soc.

[111] Barrantes S, Egea AJS, Rojas HAG (2017). Differential diagnosis between Parkinson’s disease and essential tremor using the smartphone’s accelerometer. PLoS ONE.

[112] Ware S, Knouse LE, Draz I, Enikeeva A (2022). Adjunct Proceedings of the 2022 ACM International Joint Conference on Pervasive and Ubiquitous Computing and the 2022 ACM International Symposium on Wearable Computers.

[113] Servia-Rodríguez S, Rachuri KK, Mascolo C, Rentfrow PJ, Lathia N, Sandstrom GM (2017). Proceedings of the 26th International Conference on World Wide Web.

[114] Lane ND, Lin M, Mohammod M (2014). BeWell: sensing sleep, physical activities and social interactions to promote wellbeing. Mobile Netw Appl.

[115] What are sleep disorders?. American Psychiatric Association.

[116] Thakur KT, Albanese E, Giannakopoulos P, Jette N, Linde M, Prince MJ (2016). Mental, Neurological, and Substance Use Disorders: Disease Control Priorities, Third Edition (Volume 4).

[117] Parkinson’s disease. National Institute of Neurological Disorders and Stroke.

[118] Charron E, White A, Carlston K (2023). Prospective acceptability of digital phenotyping among pregnant and parenting people with opioid use disorder: a multisite qualitative study. Front Psychiatry.

[119] Kapoor S, Narayanan A (2023). Leakage and the reproducibility crisis in machine-learning-based science. Patterns (N Y).

