# Supplementary material for: Smartphone-Based Digital Phenotyping Across Health Conditions: Scoping Review
Source: J Med Internet Res. 2026 Mar 24;28:e84146. doi: 10.2196/84146 (PMC13013828; doi:10.2196/84146)
Supplement: Multimedia Appendix 1 — Presents the full electronic search strategies used to identify relevant records. [file jmir-v28-e84146-s001.docx]

Table S1 presents the full electronic search strategies used to identify relevant records. Searches targeted peer-reviewed literature on smartphone-based digital phenotyping across health conditions. Search strings are reported exactly as executed. Searches were limited to English-language articles published between **January 1, 2012 and October 31, 2025**.

**Table S1.** Search strings used in academic databases.

| **Academic database** | **Search string (exact query)** | **Filters applied** |
| --- | --- | --- |
| PubMed | (  "Digital Phenotyping"[Title/Abstract]  OR "digital phenotype*"[Title/Abstract]  OR "mobile sensing"[Title/Abstract]  OR "smartphone sensing"[Title/Abstract]  OR "passive sensing"[Title/Abstract]  OR "personal sensing"[Title/Abstract]  )  AND  (  smartphone*[Title/Abstract]  OR "mobile phone*"[Title/Abstract]  OR "cellular phone*"[Title/Abstract]  )  AND  (  "Health"[MeSH]  OR "Mental Health"[MeSH]  OR "Disease"[MeSH]  OR health*[Title/Abstract]  OR mental*[Title/Abstract]  OR clinical*[Title/Abstract]  )  AND ("2012"[Date - Publication] : "2025"[Date - Publication])  AND English[Language] | • Language: English  • Date range: 2012–2025 |
| IEEE Xplore | ("digital phenotyping" OR "digital phenotype*" OR "mobile sensing" OR "passive sensing")  AND  (smartphone* OR "mobile phone*" OR "cell phone*")  AND  (health OR mental OR clinical OR disease OR monitoring) | • Language: English  • Content type: Journals + Conferences  • Year: 2012–2025 |
| ACM Digital Library | Abstract:(  "digital phenotyping"  OR "digital phenotype*"  OR "mobile sensing"  OR "smartphone sensing"  OR "passive sensing"  )  AND  Abstract:(smartphone* OR "mobile phone*" OR "cell phone*")  AND  Abstract:(health OR mental OR clinical OR disease) | • Language: English  • Year: 2012–2025  • Content: Journals + Proceedings |
| Google Scholar | ("digital phenotyping" OR "smartphone sensing" OR "mobile sensing" OR "personal sensing")  AND (smartphone OR "cell phone")   AND (health OR mental OR behavioral OR monitoring OR disorder OR disease) | • Custom range: 2012–2025  • Language: English  • Sorted by relevance |
